# pH effects on the electrochemical reduction of CO_(2)_ towards C_2_ products on stepped copper

**DOI:** 10.1038/s41467-018-07970-9

**Published:** 2019-01-03

**Authors:** Xinyan Liu, Philomena Schlexer, Jianping Xiao, Yongfei Ji, Lei Wang, Robert B. Sandberg, Michael Tang, Kristopher S. Brown, Hongjie Peng, Stefan Ringe, Christopher Hahn, Thomas F. Jaramillo, Jens K. Nørskov, Karen Chan

**Affiliations:** 1 SUNCAT Center for Interface Science and Catalysis, Department of Chemical Engineering, Stanford University, Stanford, California 94305 USA; 2 SUNCAT Center for Interface Science and Catalysis, SLAC National Accelerator Laboratory, 2575 Sand Hill Road, Menlo Park, California 94025 USA; 3 Institute of Natural Sciences, Westlake Institute for Advanced Study, Westlake University, Hangzhou, 310024 China; 4 Department of Chemistry and Chemical Engineering, Guangzhou University, Guangzhou, 510006 China; 5 CatTheory Center, Department of Physics, Technical University of Denmark, 2800 Kongens Lyngby, Denmark

## Abstract

We present a microkinetic model for CO_(2)_ reduction (CO_(2)_R) on Cu(211) towards C_2_ products, based on energetics estimated from an explicit solvent model. We show that the differences in both Tafel slopes and pH dependence for C_1_ vs C_2_ activity arise from differences in their multi-step mechanisms. We find the depletion in C_2_ products observed at high overpotential and high pH to arise from the 2^nd^ order dependence of C-C coupling on CO coverage, which decreases due to competition from the C_1_ pathway. We further demonstrate that CO_(2)_ reduction at a fixed pH yield similar activities, due to the facile kinetics for CO_2_ reduction to CO on Cu, which suggests C_2_ products to be favored for CO_2_R under alkaline conditions. The mechanistic insights of this work elucidate how reaction conditions can lead to significant enhancements in selectivity and activity towards higher value C_2_ products.

## Abstract (web summary)

CO_2_ conversion to reduced products provides a use for greenhouse gases, but reaction complexity stymies mechanistic studies. Here, authors present a microkinetic model for CO_2_ and CO reduction on copper, based on ab initio simulations, to elucidate pH’s impact on competitive reaction pathways.

## Introduction

Electrochemical CO_2_ reduction is a potential candidate for sustainable energy conversion and storage^1^. If this process could be realized at a reasonable efficiency and cost, fuels and basic chemicals can then be made in a sustainable way, thus allowing for a zero-emission energy conversion cycle^2–4^. In particular, the production of C_2_ products, which are usually of higher value and have higher energy densities than simpler products like H_2_ and CH_4_, is especially attractive for applications in energy storage, transportation, and the chemical industry^4,5^.

CO_2_ reduction to these products, however, presents major challenges^6,7^. Existing catalysts require large overpotentials to give significant reaction rates and the selectivity toward the desired products is often low, with hydrogen evolution being the major competing process^6,8–10^. Copper-based materials are the only catalysts that show significant selectivity toward more reduced hydrocarbons and alcohols^11,12^, but they still require high overpotentials^13–15^. The complexity of the reaction network, the importance of electrochemical activation energies^16^, and the influence of ion-adsorbate interactions also pose major challenges toward the development of a mechanistic understanding of the activity and selectivity toward C_2+_ products.

Recently, many experimental^17–25^ research efforts have been devoted to shed light on possible reaction mechanisms toward C_2_ products on Cu. In in situ-infrared spectroscopic^18,20,22,23,25^ studies, CO* has been consistently observed^20,22,23,25^, suggesting CO* to be a key reaction intermediate. Additionally, a OCCOH* signal on Cu(100) was reported during CO reduction (COR) suggestive of a CO dimerization pathway, followed by proton–electron transfer^23^. Investigations on the change of product distribution with variations in reactant feed have also been carried out to help elucidate possible pathways^17,21^. CO*^26^, glyoxal^17^, and acetaldehyde^21^, for example, have been proposed as reaction intermediates toward further reduced oxygenates using this approach. All of the above approaches, however, are limited in that the intermediates probed must be relatively stable in order to be detected spectroscopically, or exist in stable aqueous or gaseous forms to act as reaction feeds. Furthermore, observable adsorbed species may only be spectators to the dominant reaction pathways.

Computationally, various models of the electrochemical interface have recently been applied to explore the reaction mechanisms and the dominant pathways, in attempts to rationalize the overall activities^14,27,28^, the facet dependence^21,29–32^, product selectivities toward hydrocarbons and alcohols^30,33,34^, or the rates toward the formation of various C_2(+)_ products^32,35^. Despite these intensive research efforts, there has been little consensus in the literature regarding C_2(+)_ reaction pathways. For example, Luo et al.^30^ suggest that the formation of C_2_ intermediates on Cu(100) has insurmountable barriers, and that the experimentally observed production of C_2_ intermediates/products on Cu(100) surfaces occurs due to surface reconstruction, while several other studies^27,35–37^ have reported C–C coupling to be facile in the presence of solvent and ions. Furthermore, the dominant coupling step that leads up to C_2_ intermediates is also under debate. Both OC–CO dimerization^14,27,28,30,35^ and OC–CHO coupling^27,30,32^ have been suggested by various theoretical studies to dominate on Cu(100). After the formation of C_2_ intermediates, the further steps are also controversial. For instance, both Calle-Vallejo et al.^14^ and Cheng et al.^35^ have reported OCCOH* to be one of the reaction intermediates on Cu(100). However, Calle-Vallejo et al.^14^ have suggested that OCCOH* is reduced to OCC*, while Cheng et al.^35^ suggest HOCCOH* to be more favorable. The pH dependence of C_2_ product formation has been suggested to arise from rate-limiting electron transfer during CO dimerization^14,32^, but adsorbate-induced states on metals are broad (~1 eV) which give instantaneous electron transfer on the timescale of atomic motion during reaction events^36^.

The different outcomes of the various mechanistic works stem to a large extent from the theoretical challenges in determining electrochemical activation barriers, and the differences in the simplifying assumptions made^27–29,33,35,38,39^. Furthermore, certain C_2_ intermediates with large dipole moments are dramatically affected by the electric field at the interface, which adds additional complexity^36,37^. Finally, the combined effects of adsorbate–adsorbate interactions, competing reaction pathways, and adsorbate coverages require kinetic modeling to elucidate the dominant pathways, while most previous theoretical studies have emphasized reaction energetics and in some cases their correlation to onset potentials for various products^28,30,32,35^. The effect of the electrolyte pH^13,14,26,40–42^, which has dramatic effects on C_2_ activity and selectivity, also requires the consideration of kinetics.

In this work, we present a pH-dependent microkinetic model of electrochemical CO_2_ reduction kinetics over Cu(211) to C_1_ and C_2_ products, based on reaction energetics estimated via explicit-solvent^38^ simulations. With the developed kinetic model, we investigate the effects of potential and pH on the C_1_ and C_2_ product activities and selectivities. We find the simulated results to be in agreement with experimental findings:^9,26,40^ the differences in Tafel slopes between C_2_ and C_1_ products at low overpotential, the depletion of C_2_ product activity at high overpotential, the dramatic impact of pH on C_2_ and C_1_ product activity and selectivity, and the similarities in CO_2_ and CO reduction activity. We find the differences in the pH dependence between the C_2_ and C_1_ pathways to arise from differences in their rate-determining proton–electron transfer steps with water as the proton source. We also show that, given the facile kinetics for CO_2_ conversion to CO on Cu, there is little difference between the activities for CO_2_ vs. CO reduction. The original mechanistic insights supplied in this work elucidate how reaction conditions can lead to significant enhancements in selectivity and activity toward higher-value C_2_ products, which have major implications for electrolyzer design.

## Results

### Reaction pathways for CO reduction

To comprehensively model CO_(2)_ reduction, we estimated the reaction energetics for various reaction pathways using explicit- solvent simulations. According to recent experimental spectroscopic^25^ and computational work^43^, the presence of CO increases the density of highly active, low-coordinated step sites on polycrystalline copper. Given the generally lower activation barriers associated on steps vs. terraces^16,44^, the activity of steps would, from a simple consideration of the Arrhenius equation, dominate the overall activity. This idea echoes what has been found in seminal single-crystal studies of various heterogeneous reactions^45,46^. We therefore focus our analysis on the stepped Cu(211) facet, since its surface contains three-atom-wide (111) terraces separated by single-atom (100) facets at the step edges^47^, which allows us to incorporate the geometric effect of the (100) facets as well.

**Fig. 1:**
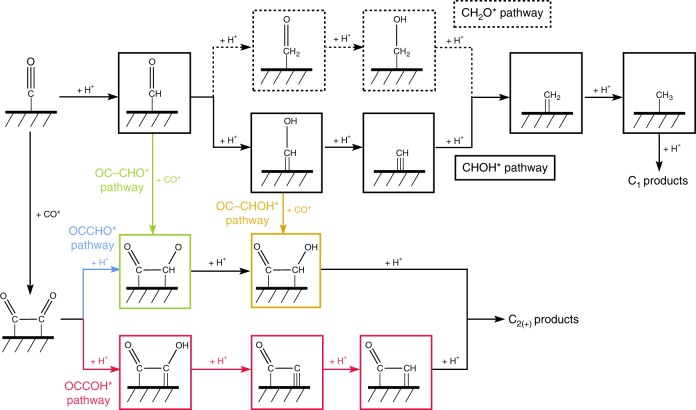
Reaction schemes of major pathways considered for CO reduction toward C_1_ and C_2+_ products. The green path denotes C_2_ production via OC–CHO coupling; the blue and red path represents C_2_ production via protonation of OCCO to form OCCHO and OCCOH, respectively; the yellow path represents C_2_ production via OC–CHOH coupling. The black path denotes C_1_ production via CHOH and the dashed CH_2_O

The main reactions considered in this work are shown in Fig. 1. As in our previous study on CO_(2)_ reduction toward C_1_ products^16^, we find the barrier for proton–electron transfer to CO* through the CHO* path to be lower than the one via COH*. The further protonation to form C_1_ products can take place via either CHOH* or CH_2_O*. For C_2_ production, we have included the coupling for CO* with various carbon species, such as CO*, CHO*, and CHOH*. As demonstrated previously^37^, the presence of a field can largely facilitate the coupling step(s), and therefore in this work, all the coupling barriers are calculated with the presence of a field. We have also assumed all the reaction steps after the formation of OCCH*, OCCHO*, and OCCHOH* to be downhill in energy. This assumption is made on the basis of a previous thermodynamic analysis which suggested all intermediates to be downhill from OCCHO*^24^, and, as discussed below, is consistent with the experimental Tafel slope which suggests an early rate-limiting proton–electron transfer. Finally, we have also included the Tafel, Heyrovsky, and Volmer elementary steps for the hydrogen evolution reaction, a major competing reaction under CO_(2)_RR conditions^9^. More calculation details and the full set of elementary reactions and the corresponding energetics can be found in the Methods section and Supplementary Information.

Despite the simplifying assumptions made, Fig. 1 shows a rather complex reaction network with many competing steps. Furthermore, the reaction is likely to take place at a substantial coverage of CO*^16^, which requires the consideration of adsorbate–adsorbate interactions. Kinetic modeling, which provides the steady-state intermediate coverages and the corresponding reaction energetics from adsorbate–adsorbate interaction models, is therefore necessary to elucidate the dominant, rate-determining steps. Trends in the calculated rates can also be directly compared with experiment. We note that the inherent uncertainties related to DFT energies^48,49^ (~0.2 eV), to the potential reference (reported values range from 4.89 to 5.17 eV)^50^ and the effects of the water structure result in larger uncertainties in electrochemical activation barriers than reaction thermochemistry. The parameterization of adsorbate–adsorbate interactions^51,52^, and the density of step-like sites in polycrystalline metal foil (estimated to be ~5%^46^, which can be used to scale our simulated curves) introduce additional uncertainties. Our focus here is therefore on a qualitative, not quantitative comparison with trends in the experiment.

### CO reduction at pH = 13

**Fig. 2:**
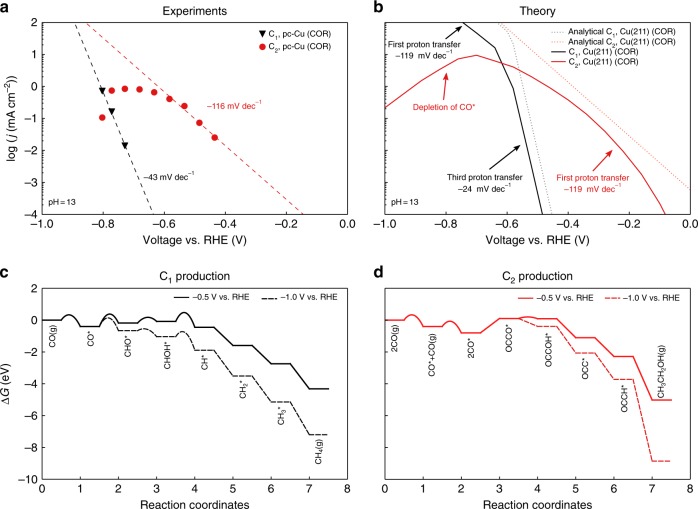
Polarization curves and free energy diagrams for C_1_ and C_2_ products on Cu. **a** Experimental polarization curves on pc-Cu of CO reduction toward C_1_ and C_2_ products at pH = 13. Data are taken from ref. ^26^. **b** Predicted polarization curves from the full microkinetic model and the associated analytical approximation on Cu(211) of CO reduction toward C_1_ and C_2_ products at pH = 13. **c** Free energy diagram for a dominant pathway at low coverage for C_1_ formation at –0.5 V and –1.0 V vs. RHE. **d** Free energy diagram for the dominant pathway at low coverage for C_2_ formation at –0.5 V and –1.0 V vs. RHE

We first examine the simple case of CO reduction at pH = 13. Figure 2a and b shows the experimentally measured^26^ and simulated polarization curves toward the formation of C_1_ and C_2_ products on polycrystalline Cu (pc-Cu) and Cu(211), respectively (see Supplementary Figure 1 and Supplementary Note 2 for polarization curves toward HER). Our description shows comparable trends to experiment, both in terms of a steeper Tafel slope for C_1_ vs. that for C_2_ products and the decrease in C_2_ activity at high overpotential. As annotated in Fig. 2a, experimentally different Tafel slopes are observed for the formation of C_1_ and C_2_ products. C_2_ formation has a Tafel slope of ~116 mV/dec, whereas C_1_ formation shows a Tafel slope of 43 mV/dec at low overpotential.

The differences in Tafel slopes arise from differences in dominant pathways. Due to the interplay between adsorbate coverages and adsorbate–adsorbate interactions, dominant pathways are not obvious from the energetics alone and must be determined through the full kinetic model. Supplementary Figure 2 shows the decomposition analysis for the various C_1_ and C_2_ pathways, and the free energy diagrams for the dominant pathways at the low coverage limit (total coverage < 0.25 ML) are shown in Fig. 2c and d (see Supplementary Figure 3 for free energy diagrams of the minor pathways). At high coverages ( > 0.25 ML) where adsorbate–adsorbate interactions set in, the barriers may increase or decrease depending on the relative strengths of interactions of the transition and initial states (see Supplementary Note 1 and Supplementary Figure 4). The influence of adsorbate–adsorbate interactions is determined by the full kinetic model self-consistently. In the potential window of interest, the model suggests the CHOH* pathway to dominate for C_1_ formation, and the OCCOH* pathway to dominate for C_2_ production, similar with some previous experimental reports on C_1_^16,34^ and C_2_^14,23,35^ formation.

We note that, due to the inherent sensitivity of rates to energetics, the uncertainties in the energetics translate to uncertainties in the decomposition analysis. The distribution of pathway contributions may also vary under different circumstances, for example, under different pH (see Supplementary Figure 2) or gas pressures. Nevertheless, as discussed below, these two pathways are sufficient to rationalize the main features of the C_1_ vs. C_2_ activity and selectivity.

**Fig. 3:**
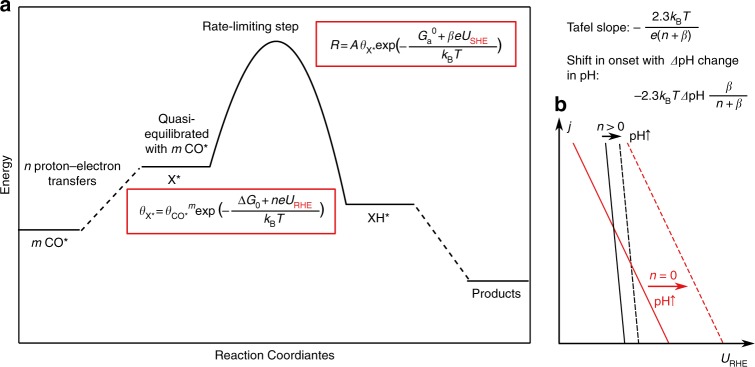
The effect of pH on multistep proton–electron transfers from H_2_O. **a** Schematic of the reaction energy landscape of a process with multiple proton–electron steps. **b** Shift in the onset with pH for reactions with *n* = 0 and *n* > 0

The Tafel slopes in the low overpotential region can be rationalized by considering the potential dependence of the rate- determining steps in the dominant pathways. As shown in Fig. 3a, in the case of a rate-determining proton–electron transfer to an adsorbate X* from water^53,54^ (i.e., alkaline or neutral conditions),

$$X^ \ast + H_2O + e^-- \to XH^ \ast + OH^-,$$

the corresponding rate of the forward reaction is given by the Butler–Volmer equation

1 $$R = A\theta _{X \ast }\exp \left( { - \frac{{G_a^0 + e\beta U_{SHE}}}{{k_BT}}} \right),$$

where *A* is the prefactor, $\theta _{{\mathrm{X}} \ast }$ the coverage of X*, $G_a^0$ the activation energy of the process at 0 V vs. SHE, *U*_SHE_ the potential vs. SHE, *β* the transfer coefficient, *k*_B_ the Boltzmann constant, and *T* the reaction temperature. *β* is a measure for the amount of charge transferred to the adsorbate at the transition state and gives the potential dependence of the barrier for the rate-determining step^40,55^. We note that the bulk pH does not play a role in *R* since neither solvated protons nor hydroxide ions are the initial or transition state in this reaction (see Supplementary Note 3 for details). The elementary reactions before the rate-determining step can be considered quasi-equilibrated, and the coverage of X is given by the following equilibrium:

$$mCO^ \ast + n\left( {H_2O + e^--} \right) \leftrightarrow X^ \ast + nOH^-,$$

where *m* = 1 or 2 depending on whether C–C coupling has occurred, *n* is the number of proton–electron transfers before the rate-limiting step, and

2 $$\theta _{X^ \ast } = \theta _{CO^ \ast }^m\exp \left( { - \frac{{\Delta G_0 + enU_{RHE}}}{{k_BT}}} \right),$$

where Δ*G*_0_ is the free energy of the process at 0 V vs. RHE, and *U*_RHE_ is the potential vs. RHE. Note that $\theta _{{\mathrm{X}} \ast }$, in this quasi-equilibrium limit, shows the Nernstian dependence on pH on the SHE scale, i.e., Eq. 2 can equivalently be written

3 $$\theta _{X^ \ast } = \theta _{CO^ \ast }^m\exp \left( { - \frac{{\Delta G_0 + enU_{SHE}}}{{k_BT}} - 2.3npH} \right).$$

The overall rate can be written either as a function of the potential relative to either RHE or SHE:

$$ R = A\theta _{{\mathrm{CO}}^ \ast }\,^m\,\exp \left( { - \frac{{G_{\mathrm{a}}^0 + \Delta G_0}}{{k_{\mathrm{B}}T}} - \frac{{n + \beta }}{{k_{\mathrm{B}}T}}\left( {eU_{{\mathrm{RHE}}} - \frac{{2.3\beta k_{\mathrm{B}}T}}{{n + \beta }}{\mathrm{pH}}} \right)} \right)$$

4 $$= A\theta _{{\mathrm{CO}}^ \ast }\,^m\,\exp \left( { - \frac{{G_a^0 + \Delta G_0}}{{k_{\mathrm{B}}T}} - \frac{{n + {\mathrm{\beta }}}}{{k_{\mathrm{B}}T}}\left( {eU_{{\mathrm{SHE}}} + \frac{{2.3nk_{\mathrm{B}}T}}{{n + \beta }}{\mathrm{pH}}} \right)} \right)$$

The Tafel slope $\frac{{\partial U}}{{\partial {\mathrm{log}}(R)}}$ is given by $- \frac{{2.3{\mathrm{k}}_{\mathrm{B}}{\mathrm{T}}}}{{\left( {n + \beta } \right)e}}$ (Fig. 3b) and therefore the later the proton transfer (and higher *n*), the steeper the corresponding slope. In the case of *n* = 0, i.e., the rate-determining step from the first proton–electron transfer to CO* or OCCO*, the rate shows a dependence only on *U*_SHE_.

The analytical approximations are shown as dotted lines in Fig. 2b. No additional barrier is found for CO dimerization, and therefore the energy difference between the adsorption energies of 2CO* and OCCO* serves as the barrier for this process. C_2_ formation is limited by the protonation of OCCO* at low overpotential, which presents an additional barrier that needs to be overcome after the formation of OCCO*. The energetic difference between *G*_TS_ of this step and 2*G*_CO*_ presents the largest barrier along this pathway (Fig. 2d). Since this is the first proton–electron transfer step (*n* *=* 0), the corresponding Tafel slope should then be 119 mV/dec, which closely resembles the experimental observation (116 mV/dec). For the production of C_1_ (Fig. 2c), on the other hand, the rate-limiting step at low overpotential is suggested to be the proton–electron transfer to CHOH*. In this case, *n* = 2, and the Tafel slope should be around 24 mV/dec. The experimental data in Fig. 2a also show a considerably smaller value than for the C_2_ products but not quite as small. We note that this discrepancy may stem from uncertainties in simulations and in the experimental mass transport limitations. In Supplementary Figure 5, we have included the effect of CO diffusion limitations, which shows that a mixed kinetic-transport region demonstrates a decreased slope for C_1_ production. In addition, Hori et al.^40^ has tested CO reduction at a series of pH from 6.0 to 12.2 and the majority of observed Tafel slopes are below 60 mV/dec (ranging from –21 to –93 mV/dec) toward C_1_ formation (Supplementary Figure 6). Nevertheless, it is evident from both Fig. 2a and Supplementary Figure 6 that C_1_ formation exhibits a steeper Tafel slope than that of C_2_ formation, which suggests that C_1_ product formation is limited by a later proton–electron transfer step at the low overpotential region.

At high overpotential, the rate-limiting step for C_1_ formation changes to the protonation of CO* (i.e., the first proton–electron transfer) and therefore the corresponding Tafel slope decreases. On the other hand, the production of C_2_ decreases, since it becomes limited by the CO* dimerization barrier and by a gradual decrease in *θ*_CO*_ (Supplementary Figure 7). C_2_ products are more severely affected by a depletion in CO* coverage than C_1_’s, since they have a second-order dependence on *θ*_CO*_. We note that, as shown in Supplementary Figure 5, CO transport limitations can also give an effective decrease in C_2_ product activity.

### The effect of pH

**Fig. 4:**
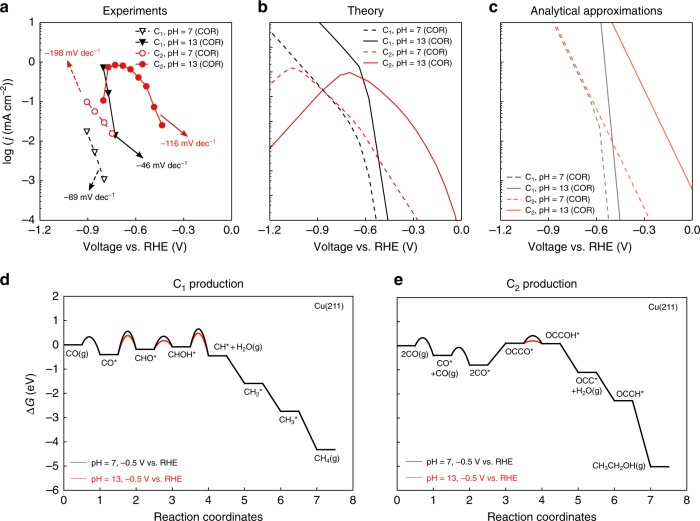
The effect of pH on C_1_ and C_2_ product activities. **a** Measured COR activities toward C_1_ and C_2_ on pc-Cu at pH = 7 and 13. Data are taken from Wang et al.^26^. **b** Predicted COR polarization curves from the microkinetic model at pH = 7 and pH = 13. **c** Approximated COR polarization curves using Eq. 4 at pH = 7 and pH = 13. **d** Free energy diagram of the dominant pathway at low coverage for C_1_ formation at –0.5 V vs. RHE at pH = 7 and pH = 13. **e** Free energy diagram of the dominant pathway at low coverage for C_2_ formation at –0.5 V vs. RHE at pH = 7 and pH = 13

The differences in rate-limiting steps for C_1_ and C_2_ formation also induce differences in pH dependence. Figure 4d and e shows corresponding variations in the free energies in the dominant pathways from a variation in pH. Reaction thermochemistry for proton–electron transfer reactions, in general, only varies as a function of *U*_RHE._ Activation energies for proton–electron transfer from H_2_O, however, remain constant at a fixed potential vs. SHE and therefore decrease at a fixed potential vs. RHE ($G_{\mathrm{a}}^{{\mathrm{H}}_2{\mathrm{O}}} = G_{{\mathrm{a}},\,0}^{{\mathrm{H}}_2{\mathrm{O}}} + \beta eU_{{\mathrm{SHE}}} = G_{{\mathrm{a}},\,0}^{{\mathrm{H}}_2{\mathrm{O}}} + \beta eU_{{\mathrm{RHE}}} - 2.3\beta k_{\mathrm{B}}T{\mathrm{pH}}$, see Supplementary Note 3 for details). The overall effect on the C_1_ vs. C_2_ activity can again be obtained by Eq. 4; the shift in overpotential with pH for a rate-determining step is given by $- 2.3k_{\mathrm{B}}T\Delta {\mathrm{pH}}\frac{\beta }{{n + \beta }}$, as shown in Fig. 3b. With $\beta \sim 0.5$, this translates to a shift of –71 mV for the C_1_ pathway (*n* = 2) and –357 mV for the C_2_ pathway (*n* = 0) between pH 7 and pH 13.

Figure 4a shows the experimental polarization curves for CO reduction reaction in a bulk pH of 7 and 13, respectively (see Supplementary Figure 1 for the HER polarization curves). Figure 4b and c shows the corresponding predictions from microkinetic modeling (Fig. 4b) and from the analytical approximation (Fig. 4c). We can see that the analytical approximation is able to give qualitatively good agreement with experiments in the low overpotential regime, giving consistent Tafel slopes and the shifts in overpotential with shifts in pH. Competing pathways present in the microkinetic model also give rise to its lower simulated rates vs. the analytical approximation, since the intermediate coverages are lower as they are consumed by multiple pathways. We have, in addition, included a comparison of our kinetic model results to the pH-dependent CO reduction data from Hori et al.^40^ in Supplementary Figure 6, as well as a comparison of HER polarization curves in Supplementary Figure 1, which also show good agreement between experimental and theoretical trends.

The experimental and theoretical pictures together show the electrolyte pH to be a way to bias the activity and selectivity toward more C_2+_ products. The ~0.36-V shift in overpotential for C_2_ products between pH 7 and 13 translates to over three orders of magnitude enhancement in C_2_ activity. The lesser shift in C_1_ products translates to a tremendous enhancement in C_2_ selectivity (1~2 order(s) of magnitude). We note that our model predicts a similar depletion in C_2_ products at high overpotential at pH 7 as in pH 13 for the same reasons, though to date no experimental data exist for pH 7 at such high overpotentials.

### CO_2_ vs. CO reduction

**Fig. 5:**
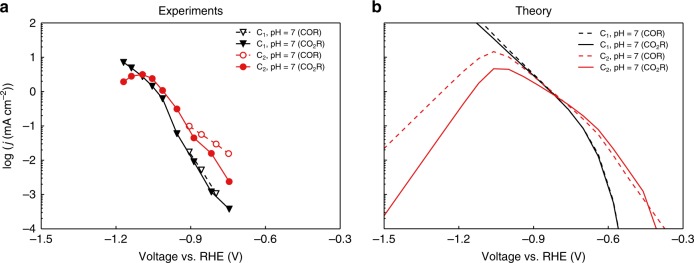
Comparison of CO and CO_2_R at pH 7. **a** Measured COR and CO_2_R activities toward C_1_ and C_2_ at pH = 7. Data are taken from refs. ^9,26^. **b** Predicted COR and CO_2_R polarization curves on Cu(211) at pH = 7

It has been suggested both experimentally^56^ and theoretically^16^ that the formation of CO* from CO_2_(g) is relatively facile on copper. CO_2_RR and COR should therefore display similar kinetics if operated at the same environmental pH. Figure 5 shows the experimentally measured (a) and simulated (b) polarization curves for both CO and CO_2_ reduction at a bulk pH of 7. The microkinetic results again exhibit good qualitative agreement with experimental trends, which places further confidence in our mechanistic understanding of the reaction. Both experimental and simulated results show the CO_2_ reduction polarization curve to coincide with the one from CO reduction in the potential window of interest. This suggests that at low/moderate overpotential, the CO* coverages are similar under COR and CO_2_R conditions (Supplementary Figure 7b), which can be rationalized by the free energy diagram (Supplementary Figure 8). Unlike gas–surface reactions, the necessary solvent reorganization adds additional barriers to gas molecule adsorption in surface electrocatalysis^57^. As shown in Supplementary Figure 8, the CO* coverage is limited by CO adsorption (*G*_a_ = 0.33 eV at low coverage) in COR and by CO_2_ adsorption (*G*_a_ = 0.45 eV at low coverage) in CO_2_R. The difference in adsorption barriers is trivial at low/moderate overpotential (*U*_RHE_ > –1.0 V), since the protonation of CO* or OCCO* are the rate-limiting steps. In this potential range, COR and CO_2_R therefore exhibit similar activities.

The difference in CO_2_ and CO adsorption barriers becomes more important at the high overpotential region (*U*_RHE_ < –1.0 V). As the proton–electron transfer barrier decreases as a function of potential, CO* formation becomes rate-limiting at high overpotential. CO* is then gradually depleted as the CO* reduction becomes increasingly favorable as potential decreases. As it is slightly more difficult to form CO* in CO_2_R, CO* is then depleted at smaller overpotentials (Supplementary Figure 7b). This effect is more pronounced in C_2_ activities, which, as discussed above, is second order in CO* coverage. Consequently, the C_2_ activities in CO_2_R decrease more rapidly at the high overpotential region, as shown in the simulated curves of Fig. 5b.

Similarly, the same trend is predicted for alkaline conditions, as illustrated in Supplementary Figure 9. This suggests that CO_2_R, if run at alkaline conditions without the CO_2_ conversion to bicarbonate, would also be expected to show a very high C_2_ activity and selectivity. We propose this simple principle to be behind a just-published work^58^, where optimized transport allowed for CO_2_R at extremely alkaline conditions, resulting in large increases in the activity and selectivity toward ethylene. This work suggested that the improved activity under alkaline conditions arises from the effect of OH* co-adsorption on the CO dimerization barrier, but our model shows no OH* coverage under reducing conditions (Supplementary Figure 7a), nor do the coverages at high potentials depend on pH on an RHE scale, since OH* adsorption is determined by reaction thermodynamics.

In summary, we presented a detailed microkinetic model of CO_(2)_ reduction on stepped Cu(211) surfaces toward C_1_ and C_2_ products. Our simulated activities show qualitative and even semiquantitative agreement with experimental observations, and we show that the distinctive potential dependence (Tafel slope, pH effects) of C_1_ and C_2_ formation can be rationalized through differences in their rate-limiting steps. C_2_ production at low overpotentials is limited by the rate of the first proton–electron transfer to the OCCO* intermediate resulting in a conventional SHE-scale dependence, while at high overpotentials, it is limited by CO coverage. C_1_ formation, on the other hand, is limited by a later proton–electron transfer to the CHOH* intermediate at low overpotential, in contrast to previous studies which focus on the protonation of CO*. Consequently, it exhibits a much higher Tafel slope and a smaller enhancement in activity with increasing pH. We also demonstrate that CO_2_R and COR show similar kinetics within the potential range of interest. The mechanistic insights supplied in this work provide ways to tune the activity and selectivity toward higher-value C_2_ products, which has major implications for the design of industrial-scale CO_(2)_R electrolyzers.

## Methods

### Computational details

Reaction energetics were calculated with density functional theory with a periodic plane-wave implementation and ultrasoft pseudopotentials using the QUANTUM ESPRESSO code^59^, interfaced with the Atomistic Simulation Environment (ASE)^60^. We applied the BEEF-vdW functional, which provides a reasonable description of van der Waals forces while maintaining an accurate prediction of chemisorption energies^61^. Plane-wave and density cutoffs were 500 and 5000 eV, respectively, with a Fermi-level smearing width of 0.1 eV.

The adsorption energies were evaluated using four-layer 3 × 3 supercells with the bottom two layers constrained, and [4 × 4 × 1] Monkhorst–Pack *k*-point grids^62^ were used. All structures were optimized until force components were less than 0.05 eV/Å. A dipole correction was applied to decouple the electrostatic interaction between the periodically repeated slabs. To determine the solvation corrections, we explicitly calculated the adsorption energy of the adsorbates in the presence of water molecules (several different configurations were considered and the lowest energy structure is taken). The solvation energy comes from the difference in adsorption energies with and without explicit water, and it arises from both the stabilization of the adsorbates through hydrogen bonding as well as the reorganization of the water layer in response to adsorption. Multiple transition metal surfaces were considered and the final correction was for simplicity taken to be the average value across all metals. Supplementary Table 1 shows the solvation corrections applied. The effect of varying the number of water layers was tested in previous work^63^. What was found was that the effect of one layer of water on the solvation of various adsorbates on rutile IrO_2_(100) was essentially equivalent to more water layers. We also considered the possibility of hydride formation, as discussed in Supplementary Note 4 and Supplementary Figure 12.

Due to GGA functionals placing the unfilled 2π* orbital too low in energy, an overbinding correction was applied to CO binding energies based on the vibrational frequency of the internal CO stretch of *CO, determined in vacuum^64,65^. The correction was 0.26 eV for Cu(211) surface. We also applied a correction of 0.15 eV per C = O double-bond correction as suggested by Christensen et al.^66^. The C = O double-bond corrections applied are listed in Supplementary Table 2. Finally, a correction of 0.33 eV was applied to the energy of CO_2_(g), which was determined from fits to experimental gas-phase reaction energetics in ref. ^67^.

Cation-induced fields lead to dramatic stabilizations of the C_2_ species involved^37^. The more degrees of freedom in the solvent in the presence of cations and solvent rearrangement in the presence of bulkier C_2_ intermediates, however, leads to higher uncertainties in the energetics. It has been suggested previously that the stabilization brought by solvation and cation-induced fields can roughly be divided^37^, and therefore we consider those two effects separately. We first obtained the field-stabilized adsorbate structures in the presence of a hydronium ion. We then applied a sawtooth potential in the *z-*direction for structures of the adsorbates in vacuum, where the solvent layer was removed. The interaction energy between an adsorbate and an electric field at the interface is given by

5 $$\Delta E = \mu {\it{\epsilon }} - \frac{1}{2}\alpha {\it{\epsilon }}^2 + \ldots$$

where Δ*E* is the change in binding energy, *ε* is the electric field strength, and *μ* and *α* are the intrinsic dipole moment and polarizability of the adsorbate, respectively^36^. We used a field strength of –0.7 V/Å to get the estimated field stabilization, as this gives us a CO* adsorption energy that is close to the value calculated with explicit solvent and field. Lastly, we added the solvation corrections as described above to obtain the adsorption energies of C_2_ species. Ongoing efforts will evaluate the energetics more rigorously by minima hopping^37,68^ the cation/solvent structures in the presence of the various intermediates.

Electrochemical barriers were calculated with (3 × 3) supercells and Monkhorst–Pack k-point grids of [4 × 4 × 1]. All structures contained a three-layer transition metal slab, with atoms in the top layer relaxed and the rest fixed, along with a hydrogen-bonded water layer determined through minima hopping^37,68^. We considered the barriers from several different water structures, the lowest of which should dominate the activity. Transition state geometries and energies were calculated using the climbing-image nudged elastic band (NEB) method, with the forces on the climbing image converged to less than 0.05 eVÅ^−1^^69^. The spring constants were tightened for images close to the saddle point^70^. The plane wave and charge density cutoff, exchange-correlation functional, and other parameters were the same as those used for geometry optimizations. The charge extrapolation method^39,55^ was used to deduce the activation barriers at constant potential^71^. All transition states were referenced to the initial state of aqueous protons and electrons in bulk solution, as determined using the computational hydrogen electrode^72^.

The water reaction pathway was modeled with an explicit water layer including a Na^+^ ion (see Supplementary Figure 10). For acidic barriers, we employed a H-down water structure as in previous work^16^ (see Supplementary Figure 10). In general, the orientation of the water structures has a significant impact on work function but not the raw, unextrapolated energies. This suggests a water-structure-dependent potential reference. We therefore, as in previous work have effectively shifted the potential reference to account for such shifts, using HER on Pt(111) as a benchmark. A + 0.8-eV shift^16^ in the potential reference for the acidic barriers gives HER polarization curves consistent with experiment^53^, as shown in Supplementary Figure 11. We note that such a shift in principle would include changes in prefactor arising from solvent reorganization, which had been considered explicitly in previous work^73^. Further details on the modeling of the hydrogen evolution are discussed in Supplementary Note 2 and Supplementary Figure 13.

### Kinetics

We took a mean-field approach to microkinetic modeling, where the net rate of an elementary reaction mA ↔ nB was given by

6 $$r = k_ + \theta _A^m--k_--\theta _B^n,$$

where *θ*_i_ represents the surface coverage of adsorbate i, *k*_+/–_ represents the rate constants of the forward and backward reaction, respectively^36^. The rate constants were calculated through the equation $k = Ae^{ - \frac{{G_{\mathrm{a}}}}{{k_{\mathrm{B}}T}}}$, where *A* represents the reaction prefactor (10^13^ s^−1^), *G*_a_ represents the activation barrier, *k*_B_ represents the Boltzmann constant, and *T* represents the reaction temperature. Site coverages were modeled using the steady-state approximation (i.e., the rate of change of all surface intermediate coverages is 0)^36^. These assumptions were implemented in the CatMAP software package^74^, which was applied to solve the microkinetic model.

Lateral adsorbate–adsorbate interactions were modeled using a first-order expansion in the coverage for the differential adsorption energy:

7 $$E_i\left( {\theta _i} \right) = E_i^0 + \mathop {\sum }\limits_j f{\it{\epsilon }}_{ij}\theta _j$$

where *E*_i_(*θ*_i_) is the differential adsorption energy of species i given a vector of coverages *θ*_i_, *E*_i_^0^ is the differential adsorption energy of species i in the low-coverage limit, *ϵ*_ij_ is a matrix of interaction parameters for the interaction between species i and j, and *f* corresponds to a piecewise-linear function for the energy as a function of coverage. The H* coverage is excluded when calculating *f* to account for H* being much smaller than CO and therefore has little effect on determining the strength of the interactions. Further information on the interaction model is provided in the former work^51^. The adsorbate cross-interaction parameters were determined using DFT calculations of the adsorption energies of intermediates at high coverages on Pt(111), and are listed in Supplementary Note 1. Further details on kinetic modeling are discussed in Supplementary Note 1 and on pH dependence in Supplementary Note 3 and Supplementary Figure 14.

### Proton versus water pathways for proton transfers

Depending on the electrolyte pH, buffer concentration, and mass transport, different proton sources will predominate. Proton transfer barriers from H_2_O or anions are generally more challenging to simulate than ones from H_3_O^+^. The larger solvation shells of OH^–^ and anions require larger model systems: whereas protons are commonly found in H_5_O_2_^+^ complexes, OH^–^ ions need 3–4 H_2_O molecules for solvation^75^. Given the small unit cell sizes used in our simulations, the work functions of the simulations with anions are generally high (~4.0–5.5 eV, corresponding to –0.4 to + 1.1 V vs. SHE), which lead to spontaneous OH^–^ adsorption on the metal slab. We also observe artificial charge transfers between the water molecules and the metal surface, due to poor band alignment between solvent and metal GGA functionals^76^.

We therefore obtained estimates for alkaline barriers from acidic calculations through several representative calculations. We investigated proton transfer barriers of HER and CO → CHO (which was found to be the rate-determining step for C_1_ products at high overpotentials^16^) with both H_3_O^+^ and H_2_O as the proton source on three transition metals that span a large range of adsorption strengths: Au, Cu, and Pt. Supplementary Figure 10 shows the structures for CO → CHO barriers calculated with both proton sources, and the comparison between acidic and alkaline barriers is shown in Supplementary Table 3. The barriers of CO → CHO are found to be similar with H_2_O or H_3_O^+^ as the proton source, while the ones of Volmer reactions are found to be on average 0.25 eV and the ones of Heyrovsky reactions are found to be on average 0.37 eV higher than their acidic counterparts. Therefore, as a first approximation, we assumed alkaline barriers for COR to be equivalent to acidic ones, and assumed Volmer barriers to be 0.25 eV higher and Heyrovsky barriers to be 0.37 eV higher than the acidic ones.

### Code availability

The CatMAP software package used in this work can be accessed and downloaded through https://github.com/SUNCAT-Center/catmap.

## Supplementary information

Supplementary Information

## Data Availability

All data generated or analyzed during this study are included in this published article (and its supplementary information files). See Supplementary Tables 4–5 for data in Fig. 2c, d, Supplementary Tables 4, 6 for data in Fig. 4d, e, and Supplementary Tables 1–8 for data to reproduce Fig. 2b, Fig. 4b, c, and Fig. 5b.
