# Supplementary material for: pH effects on the electrochemical reduction of CO(2) towards C2 products on stepped copper
Source: Nat Commun. 2019 Jan 3;10:32. doi: 10.1038/s41467-018-07970-9 (PMC6318338; doi:10.1038/s41467-018-07970-9)
Supplement: Supplementary file 1 — Supplementary Information [file 41467_2018_7970_MOESM1_ESM.pdf]

Supplementary Information for:

Liu *et al.*, pH effects on the electrochemical reduction of $CO_{(2)}$ towards C_2 products on stepped copper

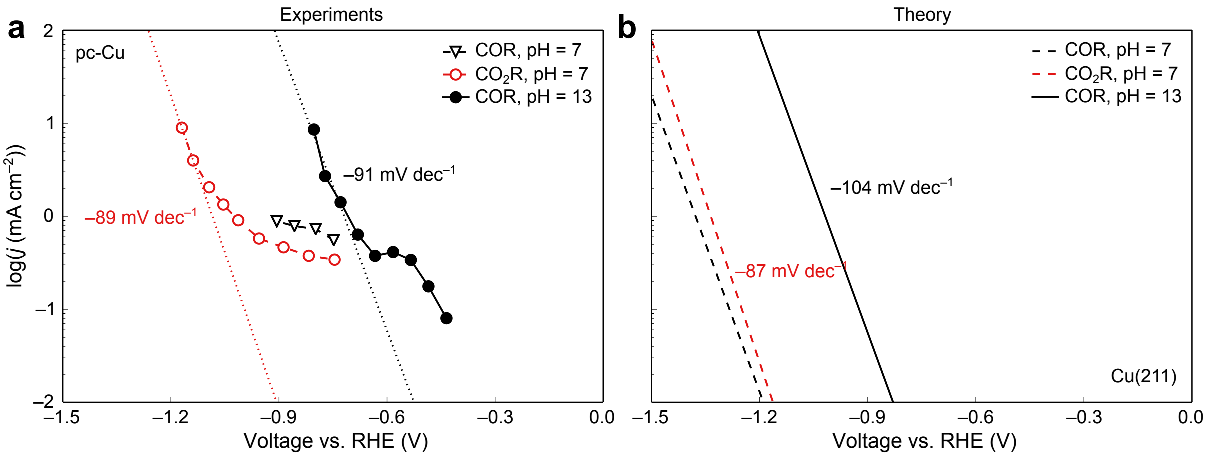

Supplementary Figure 1. HER polarization curves under neutral and alkaline conditions. (a) Experimental polarization curves on polycrystalline Cu (pc-Cu) towards hydrogen evolution reaction (HER). Data is taken from Ref. [S1, S2]; corresponding solutions are: 0.1 M $KHCO_3$ for CO_2 reduction (CO_2R) at pH = 7, 0.1 M pH 7 borate solution for CO reduction (COR) at pH = 7, and 0.1 M KOH for COR at pH = 13 (b) Predicted polarization curves from microkinetic model on Cu(211) towards HER.

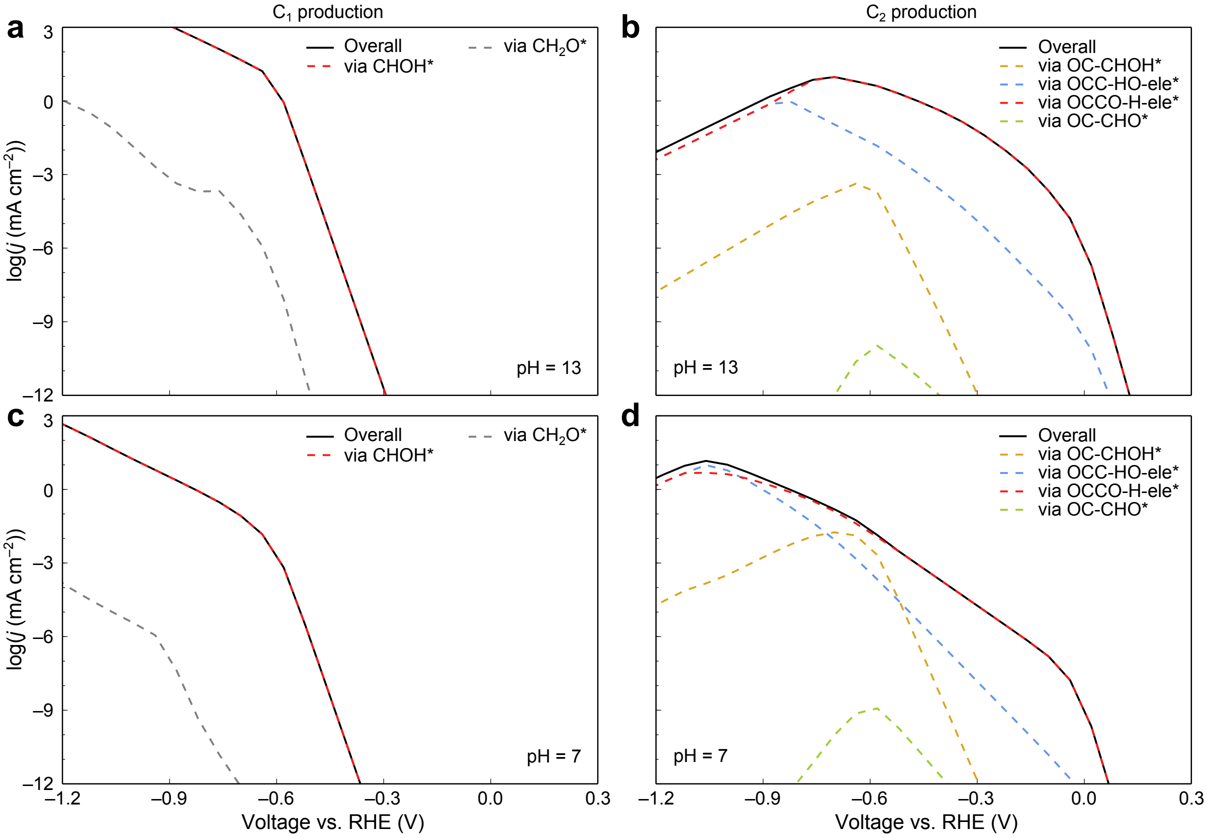

Supplementary Figure 2. Decomposition of contributions from different pathways towards C_1 and C_2 production rates at pH = 7 and 13. (a) C_1 production at pH 13. (b) C_2 production at pH 13. (c) C_1 production at pH 7. (d) C_2 production at pH 7.

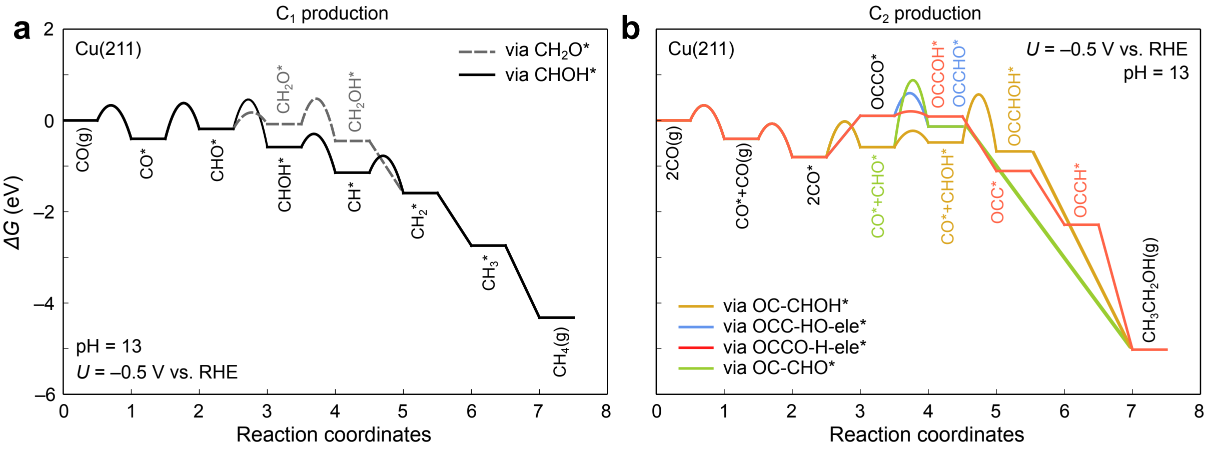

Supplementary Figure 3. Free energy diagrams of all pathways at pH = 13, *U* = –0.5 V vs. RHE at low coverage. (a) for C_1 production. (b) for C_2 production.

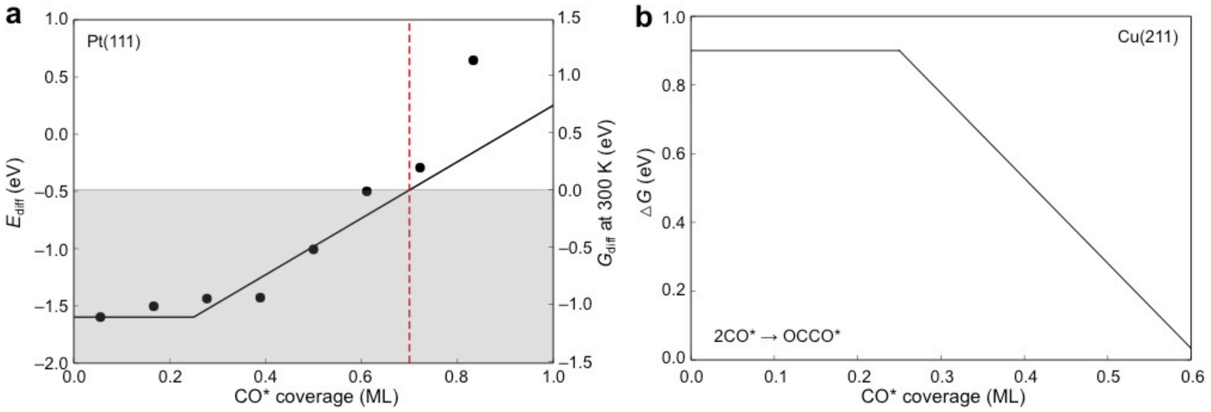

Supplementary Figure 4. Coverage dependence of CO adsorption and coupling energetics. (a) Coverage-dependent differential binding energies for CO adsorption on the Pt(111) surface. The black circles are from DFT calculations and are estimated as $E_{diff}(\theta) = \frac{E_{int}(\theta+0.5\delta)-E_{int}(\theta-0.5\delta)}{\delta}$. The solid line denotes the adsorbate-adsorbate interaction model. The red dashed line indicates the corresponding equilibrium coverage at $P_{CO(g)}$ = 1 bar. (b) Coverage-dependent reaction energy for 2CO* → OCCO* on Cu(211), as predicted by the adsorbate-adsorbate interaction model.

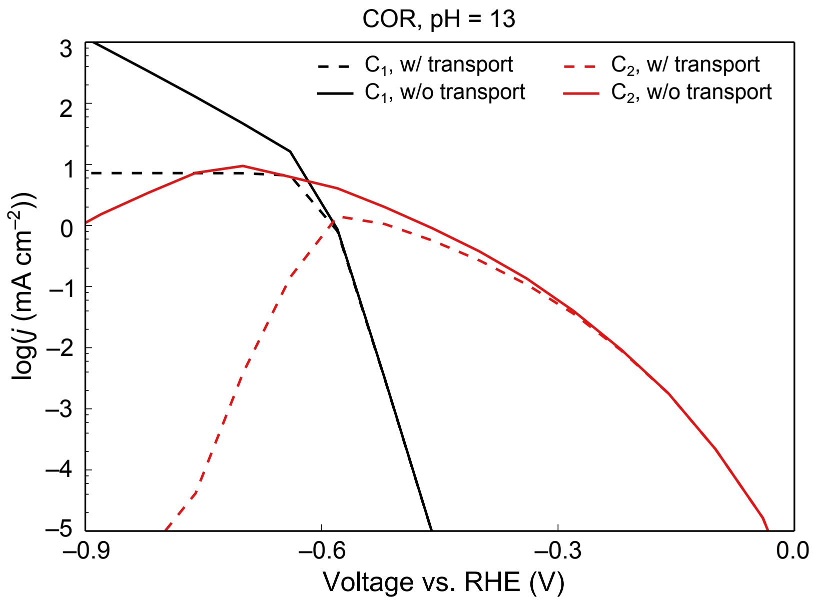

Supplementary Figure 5. Simulated COR rates from microkinetic model with and without CO(g) mass transport limitations.

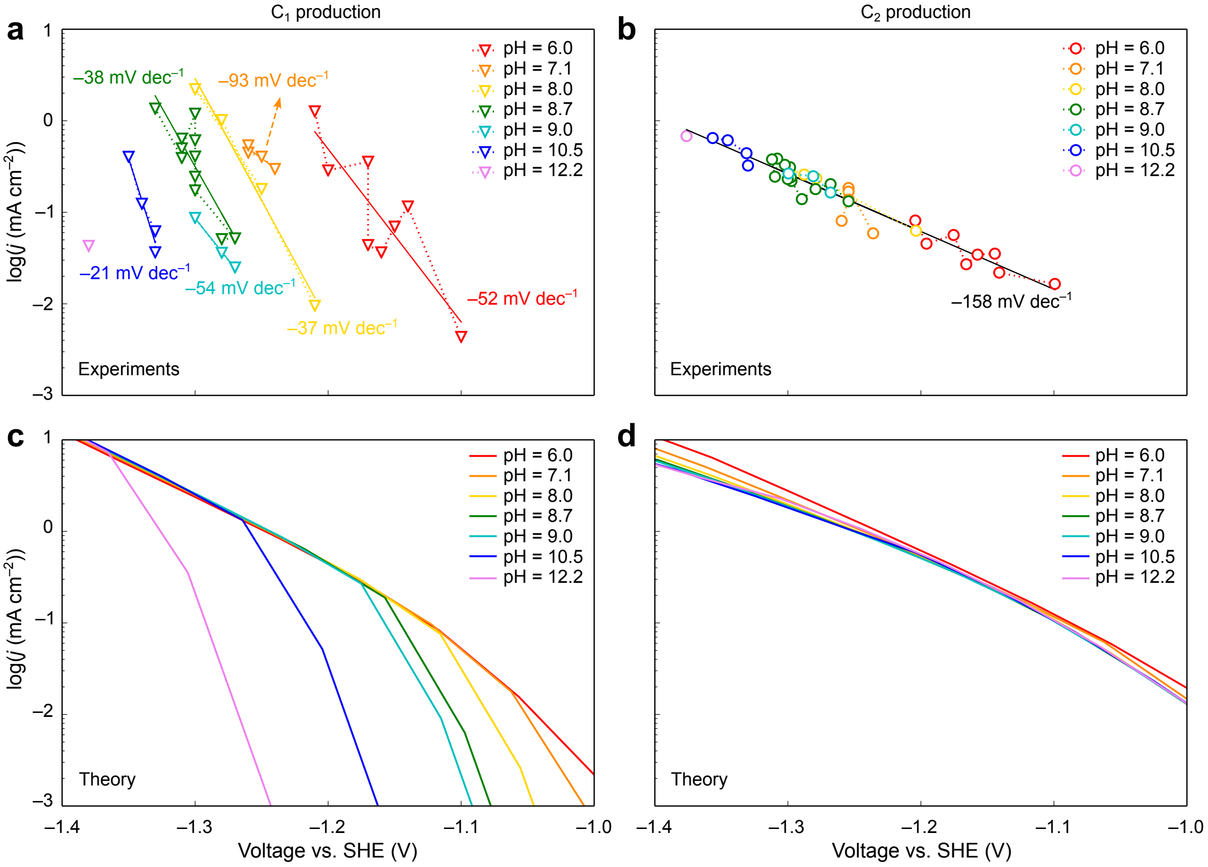

Supplementary Figure 6. Analysis of data from Ref. [S3]. (a-b) Experimentally measured COR towards C_1 products (a) and C_2 products (b) at different pH, plotted on SHE scale. Data is taken from Ref.[3] (c-d) Simulated COR rates from microkinetic model towards C_1 products (c) and C_2 products (d) at different pH, plotted on SHE scale. C_2 production shows only SHE dependence whereas C_1 production shows a mixed RHE/SHE dependence. At high overpotential, the simulation suggests that C_1 production should also show only SHE dependence. This was not observed experimentally, which can be due to the limited range in potential measured.

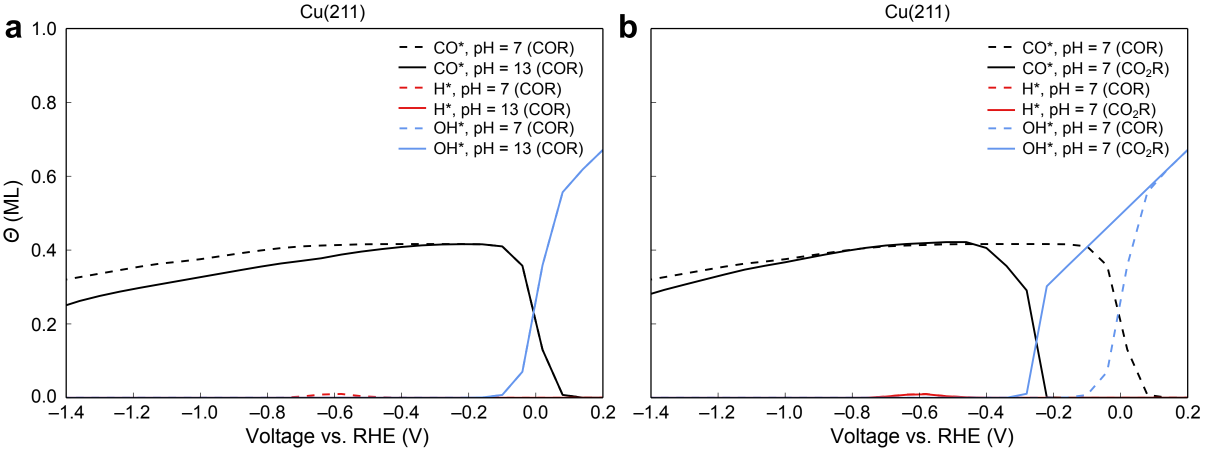

Supplementary Figure 7. Coverages of key adsorbates at various pH under COR and CO_2R conditions. (a) Surface coverages of H*, CO* and OH* for COR on Cu(211) at pH = 7 and 13. (b) Surface coverages of H*, CO* and OH* for CO and CO_2R on Cu(211) at pH = 7.

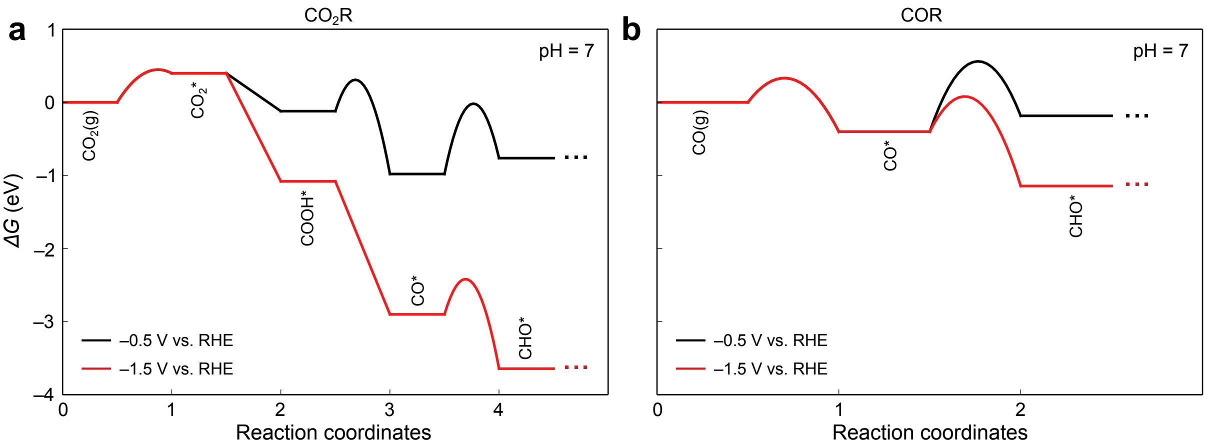

Supplementary Figure 8. Free energy diagrams towards the formation of CO* at pH = 7 at low coverage. (a) CO_2R and (b) COR.

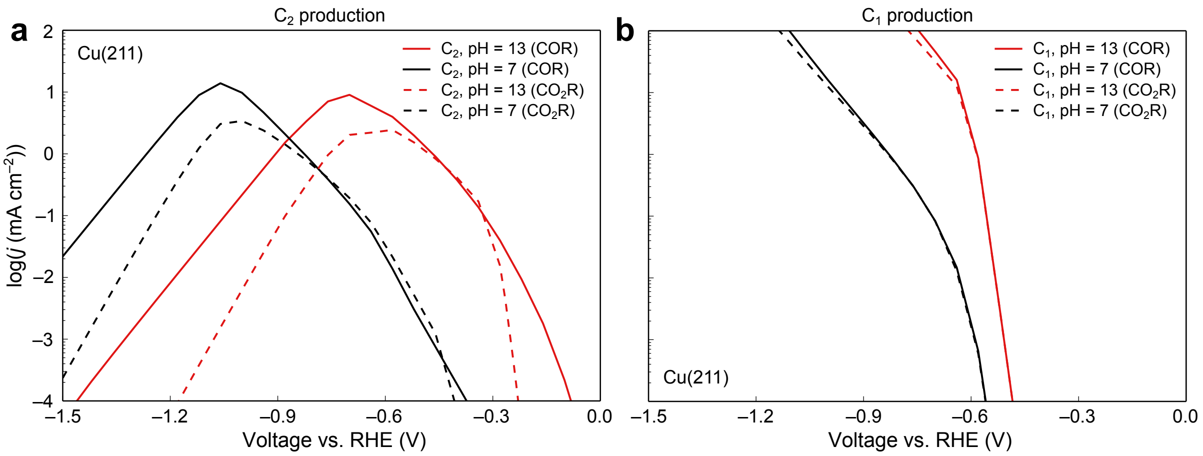

Supplementary Figure 9. Comparison of theoretical CO_2 and COR activities under neutral and alkaline conditions. (a) Simulated C_2 (b) and C_1 production rates at pH 7 and pH 13 under COR and CO_2R conditions.

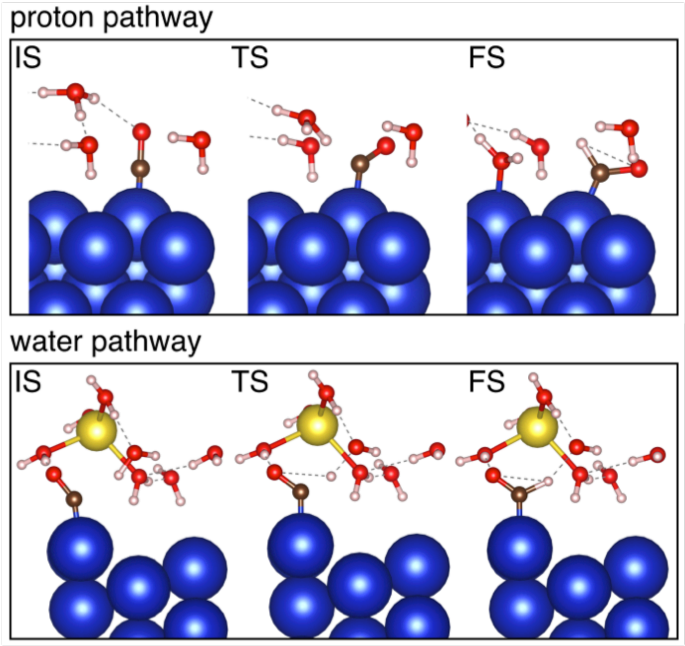

Supplementary Figure 10. Initial, transition and final state structures on Cu(211) of CO* to CHO* in proton and ion-assisted water pathway.

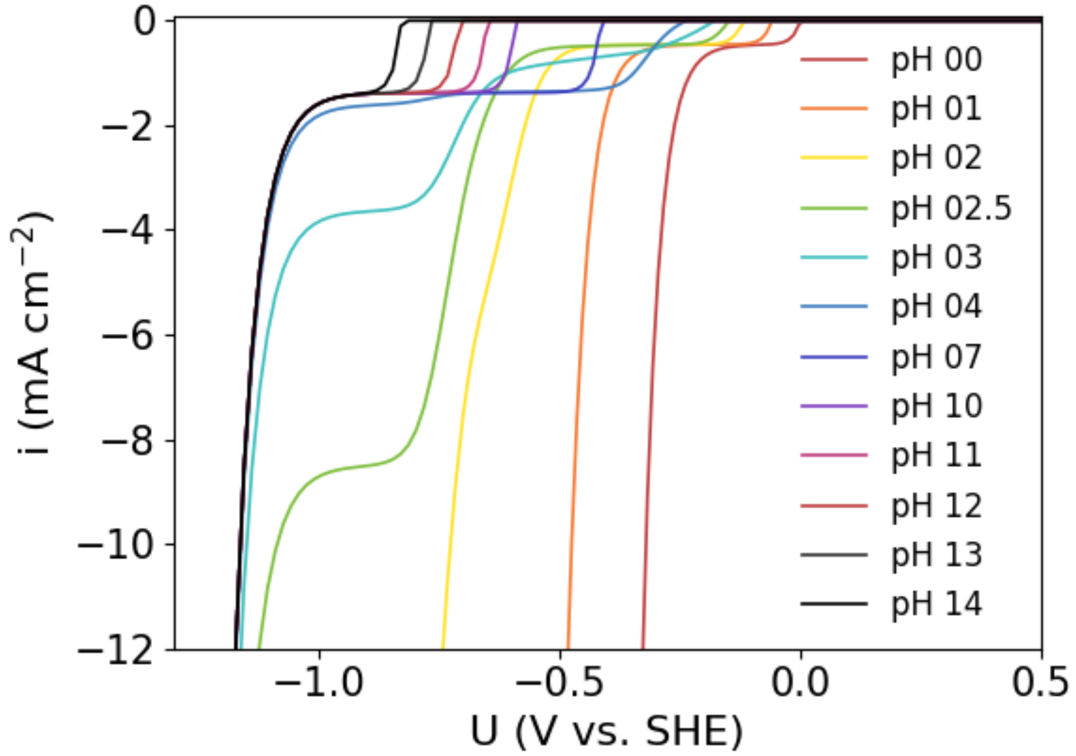

Supplementary Figure 11. pH-dependent polarization curves of HER. Curves are from our microkinetic model including H_2O and H_3O^+ proton sources, plotted on the SHE scale. The finite currents at low current densities are due to the Tafel reaction. Otherwise, the HER proceeds via Volmer-Heyrovsky.

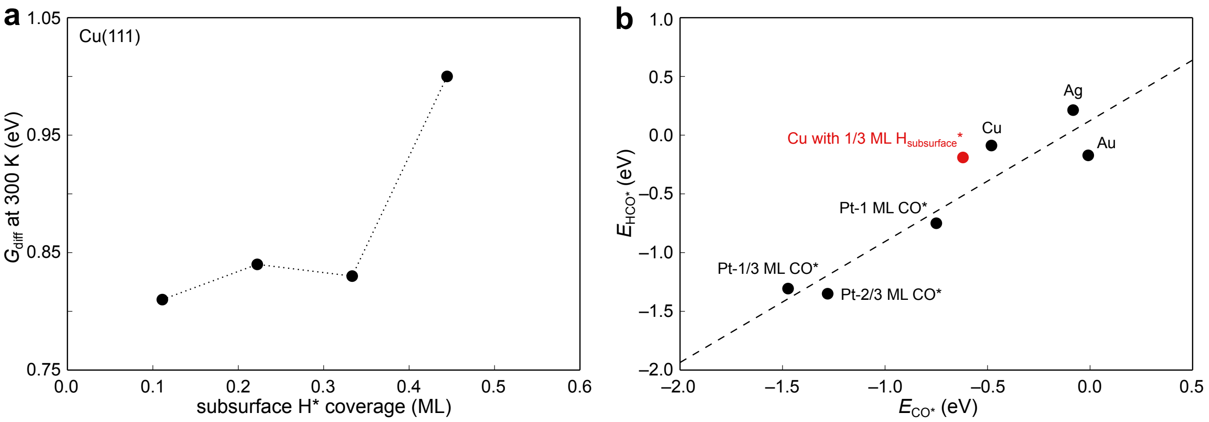

Supplementary Figure 12. Investigation of the effect of subsurface H* on Cu(111). (a) Differential subsurface H* binding energy as a function of its coverage on Cu(111). (b) Thermodynamic scaling between CHO* and CO* on transition metal (111) surfaces. Red point denotes the Cu(111) surface with 1/3 ML of subsurface H*.

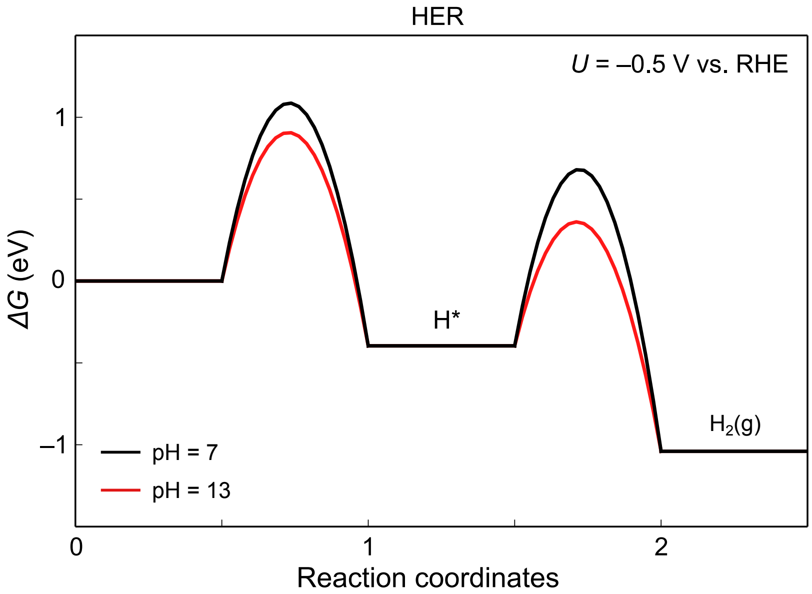

Supplementary Figure 13. Free energy diagrams towards HER under pH = 7 and pH = 13 at U_{RHE} = –0.5 V at low coverage. We note that here the data was plotted on RHE scale.

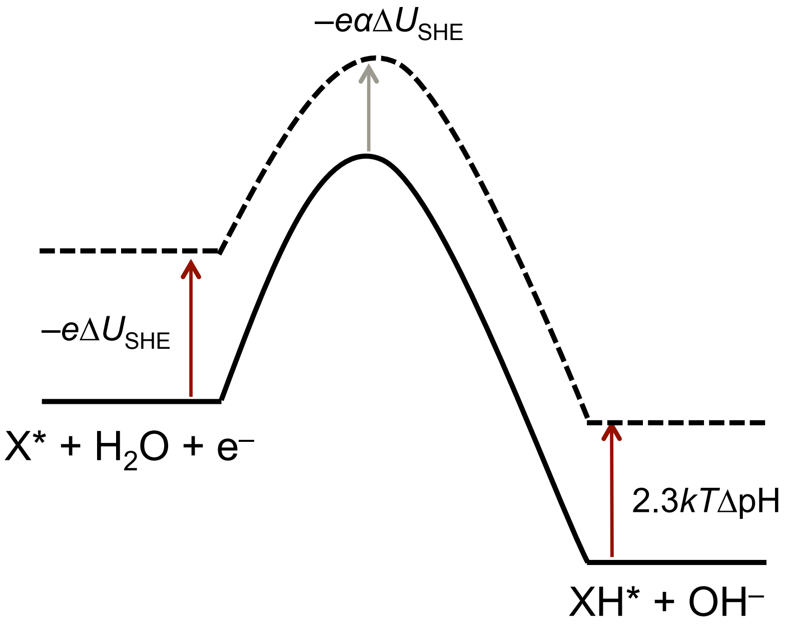

Supplementary Figure 14. Free energy diagram of alkaline proton-electron transfer reaction X* + H_2O + e^- → XH* + OH^-. Reaction energies are invariant with the potential vs. RHE and the activation barriers show dependence of potential on SHE scale.

Supplementary Table 1. Solvation corrections applied in this work.

Species	Corrections (eV)	Species	Corrections (eV)
CO^*	–0.2	$OCCHO^*$	–0.5
CHO^*	–0.3	$OCCHOH^*$	–0.45
CH_2O^*	–0.2	$OCCH^*$	–0.2
$CHOH^*$	–0.25	CO_2^*	–0.25
$OCCO^*$	–0.4	$COOH^*$	–0.25
$OCCOH^*$	–0.45	OH^*	–0.13

Supplementary Table 2. C=O double bond corrections applied in this work.

Species	Corrections (eV)	Species	Corrections (eV)
CH_2O(g)	0.15	OCC*	0.15
CHO*	0.15	OCCH*	0.15
CH_2O*	0.15	OCCHO*	0.3
OCCO*	0.3	OCCHOH*	0.15
OCCOH*	0.15	COOH*	0.25

Supplementary Table 3. Activation energies for HER and CO → CHO from different proton sources. The ones with H_3O^+ as the proton source are at 4.4 eV vs. SHE and the ones with H_2O as the proton source are at 3.63 eV vs. SHE.

Surface	HER				CO → CHO	
	Volmer reaction		Heyrovsky reaction			
	H_3O^+	H_2O	H_3O^+	H_2O	H_3O^+	H_2O
Au(211)	0.86	1.12	0.73	1.08	0.80	0.96
Cu(211)	0.87	1.10	0.79	1.17	0.73	0.81
Pt(211)	0.78	1.03	0.83	1.91	1.56	1.56

Supplementary Table 4. Adsorption free energies of the reaction intermediates used for the free energy diagrams in Figure 2 and Figure 4, in eV

Species	G_{ads} (eV)	Species	G_{ads} (eV)
CO*	–0.79	CH_3*	–0.72
CHO*	–0.08	OCCO*	–0.68
CHOH*	0.53	OCCOH*	–0.20
CH*	0.57	OCC*	–0.98
CH_2*	–0.07	OCCH*	–1.66

Supplementary Table 5. Transition state free energies used for the free energy diagrams in Figure 2 at 0 V vs. RHE at pH = 13, in eV. The corresponding activation barriers can be calculated via: $G_a = G_{TS} - G_{IS}$

Species	G_{TS} (eV)	Species	G_{TS} (eV)
H-CO-ele*	0.41	OCCO-H-ele*	-0.25
CHO-H-ele*	0.70	OCCOH-H-e*	-0.10
CHOH-H-ele*	1.51	OCC-H-ele*	-0.91

Supplementary Table 6. Transition state free energies used for the free energy diagrams in Figure 4 at 0 V vs. RHE at pH = 7, in eV. The corresponding activation barriers can be calculated via: $G_a = G_{TS} - G_{IS}$

Species	G_{TS} (eV)	Species	G_{TS} (eV)
H-CO-ele*	0.59	OCCO-H-ele*	-0.04
CHO-H-ele*	0.88	OCCOH-H-e*	0.08
CHOH-H-ele*	1.69	OCC-H-ele*	-0.73

Supplementary Table 7. Additional adsorption free energies used for the kinetics in Figure 2, 4 and Figure 5, in eV

Species	G_{ads} (eV)	Species	G_{ads} (eV)
CH_2OH*	–0.03	H*	0.10
CH_2O*	0.03	OCCHOH*	–0.46
COH*	0.95	OCCHO*	–0.42
CO_2*	–0.33	OH*	–0.23
COOH*	–0.35		

Supplementary Table 8. Additional transition state free energies used for the kinetics in Figure 2, 4 and Figure 5 at 0 V vs. SHE, in eV. The corresponding activation barriers can be calculated via: $G_a = G_{TS} - G_{IS}$

Species	G_{TS} (eV)	Species	G_{TS} (eV)
CH-H*	1.08	H-CO*	0.67
CH_2-H*	0.41	H-H*	1.00
CH_2O-H-ele*	1.13	H-ele*	1.54
CH_2O-ele*	1.38	H_2-ele*	1.90
CH_2OH-H-ele*	1.15	OC-CHOH*	0.79
CH_3-H*	0.07	OC-CHO*	0.60
CO-H-ele*	0.91	OCC-HO-ele*	0.63
CO-H*	2.11	OCC-HO*	0.42
CO_{TS}*	–0.06	OCC-H*	–0.07
COH-H-ele*	2.99	CO-OHH-ele*	0.71
CO_{2TS}*	–0.28		

Supplementary Note 1: Details of the kinetic model

Adsorbate-adsorbate interactions are considered for all possible reaction intermediate pairs. The following equations are adopted to describe the adsorption energy as a function of coverage[S4]

$$E_i(\theta_i) = \begin{cases} E_i^0 & \text{when } |\theta| \le \theta_0 \\ E_i^0 + \sum_j f\varepsilon_{ij}\theta_j & \text{when } |\theta| > \theta_0 \end{cases} \quad \text{(Equation S1)}$$

where $E_i(\theta_i)$ denotes the differential adsorption energy of species I at coverage θ_i, E_i^0 the differential adsorption energy at low coverage limit, θ_0 the threshold coverage (0.25 ML in this work), $|\theta|$ the sum of the surface coverages of all adsorbates except H* (the H* coverage is excluded to account for H* being much smaller than CO and therefore has little effect on determining the strength of the interactions), ε_{ij} the cross-interaction parameter between species I and j, f the fractional coverage, which can be calculated as $f = \frac{|\theta|-\theta_0}{|\theta|}$.

As shown above, the interactions are significant only when adsorbate coverages exceed a threshold of about 0.25 monolayers[S4], and CO* is the only intermediate that has a coverage above this threshold in the potential range of interest, therefore only the interactions between CO* and other intermediates affect the energetics. Supplementary Figure 4(a) shows, as an example, the coverage dependence of CO adsorption on Pt(111). The energetics of intermediates and transition states are therefore all functions of coverage. The barriers and reaction energies could then be higher or lower at high surface coverage. For example, as shown in Supplementary Figure 4(b), the reaction energy of 2CO* → OCCO* decreases as CO* coverage increases, since the interaction parameter $\epsilon_{CO*,\ OCCO*}$ = 2.47 is smaller than 2×$\epsilon_{CO*,\ CO*}$ = 4.93. We have explicitly parameterized the CO self-interaction and its cross-interaction parameters with the species in the rate-limiting steps. For the other intermediates that are not in the rate-limiting steps, their interaction parameters do not change the kinetic results significantly. We therefore have assumed the interaction parameters to be the same due to the similar sizes of molecules.

The adsorbate cross-interaction parameters were listed below.

$\epsilon_{CO*,\ CO*}$ = 2.47

$\epsilon_{CO*,\ H*}$ = 0.73

$\epsilon_{CO*,\ H\text{-}H*}$ = 1.16

$\epsilon_{CO*,\ H\text{-}ele*}$ = 0.79

$\epsilon_{CO*,\ H2\text{-}ele*}$ = 0.51

$\epsilon_{CO*,\ H\text{-}CO\text{-}ele*}$ = 1.95

$\epsilon_{CO*,\ CO\text{-}H\text{-}ele*}$ = 7.87

$\epsilon_{CO*,\ H\text{-}COH\text{-}ele*}$ = 1.38

$\epsilon_{CO*,\ CO2*}$ = $\epsilon_{CO*,\ CO2\_TS*}$ = $\epsilon_{CO*,\ CO\text{-}OHH\text{-}ele*}$ = 1.48

$\epsilon_{CO*,\ H\text{-}CO\ *}$ = $\epsilon_{CO*,\ CO\text{-}H*}$ = $\epsilon_{CO*,\ CH\text{-}H*}$ = $\epsilon_{CO*,\ CH2\text{-}H*}$ = $\epsilon_{CO*,\ CH3\text{-}H*}$ = $\epsilon_{CO*,\ CH2O\text{-}ele*}$ = $\epsilon_{CO*,\ CH2O\text{-}H\text{-}ele*}$ = $\epsilon_{CO*,\ CH2OH\text{-}H\text{-}ele*}$ = $\epsilon_{CO*,\ OC\text{-}CO*}$ = $\epsilon_{CO*,\ OC\text{-}CHO*}$ = $\epsilon_{CO*,\ OC\text{-}CHOH*}$ = $\epsilon_{CO*,\ OCCO\text{-}H*}$ = $\epsilon_{CO*,\ OCCO\text{-}H\text{-}ele*}$ = $\epsilon_{CO*,\ OCCOH\text{-}H\text{-}ele*}$ = $\epsilon_{CO*,\ OCC\text{-}H\text{-}ele*}$ = $\epsilon_{CO*,\ OCC\text{-}H*}$ = $\epsilon_{CO*,\ OCC\text{-}HO*}$ = $\epsilon_{CO*,\ OCC\text{-}HO\text{-}ele*}$ = $\epsilon_{CO*,\ OC\text{-}CH2O*}$ = $\epsilon_{CO*,\ COTS*}$ = $\epsilon_{CO*,\ COOH*}$ = 2.47

$\epsilon_{OH*,\ OH*}$ = 1.03

CH^*, CH_2^*, CH_3^*, $CHOH^*$, CHO^*, COH^*, CH_2O^*, $OCCO^*$, $OCCHO^*$, $OCCOH^*$, OCC^*, $OCCH^*$, $OCCHOH^*$, $OCCH_2O^*$ were assumed to have same interactions as those of CO^*. All unlisted $\epsilon_{I,j}$ are assumed to be zero.

In this simple model, $CH_4(g)$ was taken as an example of C_1 products, $CH_3CH_2OH(g)$ was taken as an example of C_2 products, and $H_2(g)$ was included as the main side product. We note that as mentioned in the main text, we have assumed all the reaction steps after the formation of $OCCH^*$, $OCCHO^*$ and $OCCHOH^*$ to be downhill in energy. And therefore the results will remain unchanged whether the example C_2 product is chosen to be ethanol or ethene. Both proton-electron transfer and surface hydrogenation pathways were taken into account. All the elementary steps are described as follows:

$H^+ + e^- + * \leftrightarrow H^*$

$H^+ + e^- + H^* \leftrightarrow H_2(g) + *$

$H^* + H^* \leftrightarrow H_2(g) + 2*$

$OH^* + H^+ + e^- \leftrightarrow H_2O(g) + *$

$CO(g) + * \leftrightarrow CO_{TS}^* \leftrightarrow CO^*$

$CO^* + H^+ + e^- \leftrightarrow CHO^*$

$CO^* + H^* \leftrightarrow CHO^* + *$

$CHO^* + H^+ + e^- \leftrightarrow CHOH^*$

$CO^* + H^+ + e^- \leftrightarrow COH^*$

$CO^* + H^* \leftrightarrow COH^* + *$

$COH^* + H^+ + e^- \leftrightarrow CHOH^*$

$CHOH^* + H^+ + e^- \leftrightarrow CH^* + H_2O(g)$

$CHO^* + H^+ + e^- \leftrightarrow CH_2O\text{-ele}^* \leftrightarrow CH_2O^*$

$CH_2O^* + H^+ + e^- \leftrightarrow CH_2O\text{-H-ele}^* \leftrightarrow CH_2OH^*$

$CH_2OH^* + H^+ + e^- \leftrightarrow CH_2OH\text{-H-ele}^* \leftrightarrow CH_2^* + H_2O(g)$

$2CO^* \leftrightarrow OCCO^*$

$OCCO^* + H^* \leftrightarrow OCC\text{-HO} + * \leftrightarrow OCCHO^* + *$

$OCCO^* + H^+ + e^- \leftrightarrow OCC\text{-HO-ele}^* \leftrightarrow OCCHO^*$

$CO^* + CHO^* \leftrightarrow OC\text{-CHO}^* \leftrightarrow OCCHO^*$

$OCCHO^* + 7H^+ + 7e^- \leftrightarrow CH_3CH_2OH(g) + 2* + H_2O(g)$

$OCCO^* + H^+ + e^- \leftrightarrow OCCO\text{-H-ele}^* \leftrightarrow OCCOH^*$

$OCCOH^* + H^+ + e^- \leftrightarrow OCCOH\text{-H-ele}^* \leftrightarrow OCC^* + H_2O(g)$

$OCC^* + H^+ + e^- \leftrightarrow OCC\text{-H-ele}^* \leftrightarrow OCCH^*$

$OCC^* + H^* \leftrightarrow OCC\text{-H}^* + * \leftrightarrow OCCH^* + *$

$OCCH^* + 5H^+ + 5e^- \leftrightarrow CH_3CH_2OH(g) + 2*$

$CO^* + CHOH^* \leftrightarrow OC\text{-CHOH}^* \leftrightarrow OCCHOH^*$

$OCCHOH^* + 6H^+ + 6e^- \leftrightarrow CH_3CH_2OH(g) + 2* + H_2O(g)$

$CH^* + H^+ + e^- \leftrightarrow CH_2^*$

$CH^* + H^* \leftrightarrow CH_2^* + *$

$CH_2^* + H^+ + e^- \leftrightarrow CH_3^*$

$CH_2^* + H^* \leftrightarrow CH_3^* + *$

$CH_3^* + H^+ + e^- \leftrightarrow CH_4(g) + *$

$CH_3^* + H^* \leftrightarrow CH_4(g) + 2*$

$CH_2OH^* + H_2O(g) + {}^*dl \leftrightarrow CH_2(OH)_2{}^*dl + * + H^+ + e^-$

$CH_2O^* + {}^*dl \leftrightarrow CH_2O\text{-}{}^*dl + * \leftrightarrow CH_2O^*dl + *$

$CH_2O^*dl \leftrightarrow CH_2O(g) + {}^*dl$

$CH_2O^*dl + H_2O(g) \leftrightarrow CH_2(OH)_2{}^*dl$

$CH_2(OH)_2{}^*dl \leftrightarrow CH_2(OH)_2(g) + {}^*dl$

In the CO_2R model, the following steps are included in addition:

$CO_2(g) + 2^* \leftrightarrow CO_{2TS}^* \leftrightarrow CO_2^*$

$CO_2^* + H^+ + e^- \leftrightarrow COOH^*$

$COOH^* + H^+ + e^- \leftrightarrow CO\text{-}OHH\text{-}ele^* \leftrightarrow CO^* + H_2O(g) + *$

where * represents a surface site, and *dl refers to the species at the double layer. All the C_2 species are assumed to take two surface sites. All the steps have prefactors of 10^{13} s^{-1} based on harmonic transition state theory[S5].

To simulate CO mass transport limitations, we have adopted similar model as described in Ref.[S6]. We have assumed that CO diffuses through a boundary layer of thickness, L, outside which convection dominates. The CO consumption rate equals the flux of CO under the steady-state assumption. According to Fick's first law, the CO flux is

$$J_{CO} = -D_{CO}\frac{\partial c_{CO}}{\partial z} \approx -D_{CO}\frac{c_{CO(\mathrm{dl})} - c_{CO(\mathrm{bulk})}}{L} \qquad \text{(Equation S2)}$$

where D_{CO} is the diffusivity of CO, $c_{CO(dl)/(bulk)}$ the CO concentration at the double layer and bulk respectively, L the thickness of double layer. According to Henry's law, the concentration of CO can be expressed as

$$c_{CO} = P_{CO}k_H \qquad \text{(Equation S3)}$$

where k_H is the Henry's constant and P_{CO} is the CO pressure. The CO flux therefore could be expressed as

$$J_{CO} = -\frac{D_{CO}}{L}k_H(P_{CO(\text{effective at dl})} - P_{CO(\mathrm{g})}) \qquad \text{(Equation S4)}$$

As the reaction rate equals CO flux, this gives

$$R = \frac{J_{CO}N_A}{\#\text{ of sites/Area}} = -N_A\frac{\text{Area}}{\#\text{ of sites}}\frac{D_{CO}}{L}k_H(P_{CO(\text{effective at dl})} - P_{CO(\mathrm{g})}) \qquad \text{(Equation S5)}$$

where N_A is the Avogadro constant. This expression shows that CO diffusion can be approximated as a chemical step with rate constants

$$k_f = k_b = N_A\frac{\text{Area}}{\#\text{ of sites}}\frac{D_{CO}}{L}k_H \qquad \text{(Equation S6)}$$

Assume a site density of 12/(7.8×9.1) Å^{-2} and a double layer thickness of 10 μm, the effective rate constant is 15. Therefore, the following steps are added to model transport limitations:

CO(g) + *dl ↔ CO*dl (k = 15)

CO*dl + * ↔ CO_{TS}* + *dl ↔ CO* + *dl

where CO*dl has the same free energy as CO(g).

Supplementary Note 2: The hydrogen evolution reaction

The experimentally measured and simulated polarization curves towards HER are shown in Supplementary Figure 1. In general the current kinetic model suggests: 1) CO_2 and COR at pH 7 to show similar HER activity (consistent with the similarity in CO and H coverages between the two conditions) 2) a ~0.36 V shift in onset for HER under COR conditions at pH 13. This SHE dependence in HER activity arises from a rate limiting Volmer step, which is first proton-electron transfer (see Supplementary Figure 13 for the corresponding free energy diagram, and refer to Equation (3) in the main text regarding SHE dependence for n=0). The model captures the experimental observations in the high overpotential region for CO_2R at pH 7 and COR at pH 13; no high overpotential data for COR at pH 7 exists. In this region, the experimental and simulated polarization curves exhibit similar Tafel slopes. At moderate overpotentials, a plateau is observed for the pH 7 data; we speculate that this plateau arises from HER from different proton donors (H_3O^+, $B(OH)_3^-$, HCO_3^-), which is not considered in the model. Ongoing work seeks to elucidate the effect of alternative proton donors on $CO_{(2)}$R and HER kinetics.

Supplementary Note 3: The pH and potential dependence

As shown in Supplementary Figure 14, for a proton-electron transfer reaction with water as the proton source, the pH affects the chemical potential of OH^- through its configurational entropy, while the absolute potential (i.e. U_{SHE}) affects the chemical potential of the electron, e^-. As the transition state is assumed to have no entropic contributions, its energy only depends on potential via transfer coefficient α that is fractional and does not depend on pH. In this case, the activation energy and the reaction energy can be expressed as

$$G_a^{\mathrm{H_2O}} = \mu_{\mathrm{TS}}+\mu_{\alpha e^-,U} - \left(\mu_{\mathrm{H_2O}} + \mu_{e^-,U} + \mu_{\mathrm{X*}}\right)$$

$$= \mu_{\mathrm{TS}}+(\mu_{\alpha e^-,0} - \alpha e U_{\mathrm{SHE}}) - \left(\mu_{\mathrm{H_2O}} + \left(\mu_{e^-,0} - eU_{\mathrm{SHE}}\right) + \mu_{\mathrm{X*}}\right)$$

$$= G_{a,0}^{\mathrm{H_2O}} + \beta e U_{\mathrm{SHE}} \qquad \text{(Equation S7)}$$

$$= G_{a,0}^{\mathrm{H_2O}} + \beta e U_{\mathrm{RHE}} - \beta 2.3 k_{\mathrm{B}} T \mathrm{pH} \qquad \text{(Equation S8)}$$

$$\Delta G^{\mathrm{H_2O}} = \mu_{\mathrm{XH*}} + \mu OH — \mu_{\mathrm{H_2O}} + \mu_{e^-,U} + \mu_{\mathrm{X*}}$$

$$= \mu_{\mathrm{XH*}} + (\mu_{\mathrm{OH^-}}^0 - 2.3 k_{\mathrm{B}} T(14 - \mathrm{pH})) - \left(\mu_{\mathrm{H_2O}} + \left(\mu_{e^-,0} - eU_{\mathrm{SHE}}\right) + \mu_{\mathrm{X*}}\right)$$

$$= \Delta G_0^{\mathrm{H_2O}} + eU_{\mathrm{SHE}} + 2.3 k_{\mathrm{B}} T \mathrm{pH} \qquad \text{(Equation S9)}$$

$$= \Delta G_0^{\mathrm{H_2O}} + eU_{\mathrm{RHE}} \qquad \text{(Equation S10)}$$

where $\mu_{\mathrm{OH^-}}^0$ denote the chemical potentials under the standard conditions, $\mu_{\mathrm{X*}}$ the chemical potential of species X*, $G_{a,0}^{\mathrm{H_2O}}$ / $\Delta G_0^{\mathrm{H_2O}}$ the activation energy and reaction energy at 0 V, α the charge in the transition state that gives the potential dependence ($\beta = 1 - \alpha$), T the temperature and k_B the Boltzmann constant. According to the equations above, reaction energies show an

RHE dependence (Equation S10), since shifts in pH are by definition balanced by shifts in absolute potential (i.e. $2.3k_BT\Delta pH = -e\Delta U$). Activation energies, however, are affected differently. At a fixed potential vs. SHE (Supplementary Figure 14, Equation S7), activation energies for proton transfer from water are unaffected by pH shifts, and decrease with increases in pH at a fixed potential vs. RHE (Equation S8).

Supplementary Note 4: Impact of hydroxide and hydride species

To evaluate the possibility that hydroxides or hydrides affect the activity, we have included *OH adsorption in the kinetic model, and determined the stability of subsurface H*. We find the OH* coverage to quickly decrease to zero at around –0.2 V vs. RHE (Supplementary Figure 7). Hydride formation on Cu(111) was investigated as well as an example. As shown in Supplementary Figure 12(a), subsurface H* is stable only at very high overpotentials. Under highly reducing conditions (e.g. $U_{RHE} < -0.85$ V), which is close to the limit of the potential window we are interested in, we find the subsurface coverage of H on Cu to be only around 1/3 ML. At this coverage of subsurface H*, the adsorption energies of the intermediates CO* and CHO* shift by only ~0.1 eV and still follow the scaling line (Supplementary Figure 12(b)). This suggests that OH* and subsurface H* formation do not have a significant effect on the energetics and the qualitative trends we are investigating.

Supplementary References

S1. Wang, L. et al. Electrochemical carbon monoxide reduction on polycrystalline copper: Effects of potential, pressure, and pH on selectivity toward multicarbon and oxygenated products. *ACS Catal.* **8**, 7445-7454 (2018).

S2. Kuhl, K. P. et al. Electrocatalytic conversion of carbon dioxide to methane and methanol on transition metal surfaces. *J. Am. Chem. Soc.* **136**, 14107-14113 (2014).

S3. Hori, Y., Takahashi, R., Yoshinami, Y. & Murata, A. Electrochemical reduction of CO at a copper electrode. *J. Phys. Chem. B* **101**, 7075-7081 (1997).

S4. Lausche, A. C. et al. On the effect of coverage-dependent adsorbate-adsorbate interactions for CO methanation on transition metal surfaces. *J. Catal.* **307**, 275-282 (2013).

S5. Nørskov, J. K., Studt, F., Abild-Pedersen, F. & Bligaard, T. Fundamental concepts in heterogeneous catalysis. (2014).

S6. Hansen, H. A., Viswanathan, V. & Nørskov, J. K. Unifying kinetic and thermodynamic analysis of 2 e^- and 4 e^- reduction of oxygen on metal surfaces. *J. Phys. Chem. C* **118**, 6706-6718 (2014).
